# Aberrant methylation of WD‐repeat protein 41 contributes to tumour progression in triple‐negative breast cancer

**DOI:** 10.1111/jcmm.15344

**Published:** 2020-05-12

**Authors:** Han Wang, Dan Wu, Liangliang Cai, Xiaohong Li, Zhiming Zhang, Shuai Chen

**Affiliations:** ^1^ Department of oncology Xiamen Fifth hospital Xiamen China; ^2^ Translational Medicine Research Center (TMRC) School of Pharmaceutical Science Xiamen University Xiamen Fujian China; ^3^ Department of Otolaryngology‐Head and Neck Surgery The First Affiliated Hospital of Xiamen University Xiamen China; ^4^ Xiamen Key Laboratory of Otolaryngology‐Head and Neck Surgery Xiamen China; ^5^ Department of Breast Surgery The First Affiliated Hospital of Xiamen University Xiamen China; ^6^ Department of Medical Oncology Cancer Hospital The First Affiliated Hospital of Xiamen University Xiamen China

**Keywords:** AKT/GSK‐3β/β‐catenin pathway, methylation, triple‐negative breast cancer, WDR41

## Abstract

WD‐repeat proteins are implicated in a variety of biological functions, most recently in oncogenesis. However, the underlying function of WD‐repeat protein 41 (WDR41) in tumorigenesis remains elusive. The present study was aimed to explore the role of WDR41 in breast cancer. Combined with Western blotting and immunohistochemistry, the results showed that WDR41 was expressed at low levels in breast cancer, especially in triple‐negative breast cancer (TNBC). Using methylation‐specific PCR (MSP), we observed that WDR41 presented hypermethylation in MDA‐MB‐231 cells. Methylation inhibitor 5‐aza‐2′‐deoxycytidine (5‐aza‐dC) management increased the expression of WDR41 in MDA‐MB‐231 cells, but not in MCF‐10A (normal mammary epithelial cells) or oestrogen receptor‐positive MCF‐7 breast cancer cells. WDR41‐down‐regulation promoted, while WDR41‐up‐regulation inhibited the tumour characteristics of TNBC cells including cell viability, cell cycle and migration. Further, WDR41‐up‐regulation dramatically suppressed tumour growth in vivo. Mechanistically, WDR41 protein ablation activated, while WDR41‐up‐regulation repressed the AKT/GSK‐3β pathway and the subsequent nuclear activation of β‐catenin in MDA‐MB‐231 cells, and 5‐aza‐dC treatment enhanced this effect. After treatment with the AKT inhibitor MK‐2206, WDR41‐down‐regulation‐mediated activation of the GSK‐3β/β‐catenin signalling was robustly abolished. Collectively, methylated WDR41 in MDA‐MB‐231 cells promotes tumorigenesis through positively regulating the AKT/GSK‐3β/β‐catenin pathway, thus providing an important foundation for treating TNBC.

## 1 INTRODUCTION

The WD40‐repeat is a short structural domain protein consisting of 40 amino acids that starts from glycine and histidine and ends with tryptophan‐aspartic acid (W‐D) dipeptide.^1^ WD40‐repeat proteins are a large family of proteins found in all eukaryotes and are involved in various cellular biological processes, including signal transduction, transcription regulation, cell cycle control, autophagy and apoptosis.^2^ Structural studies indicate that the WD40‐repeat proteins have scaffolding capability, thus mediating diverse protein‐protein interactions and critical cellular functions.^3^

WD‐repeat proteins are well known as tumour biomarkers and are highly expressed in most types of cancer.^4^, ^5^ The WD‐repeat protein 16 (WDR16 or WDRPUH), containing 11 highly conserved WD40‐repeat domains, is associated with the 70‐kD heat shock protein (HSP70; proteins of the chaperonin‐containing TCP‐1 complex) and breast cancer type 2 susceptibility protein, and participates in the tumorigenesis of hepatocellular carcinoma.^6^ Increased expression of constitutive photomorphogenic 1 (COP1, also known as E3 ubiquitin protein ligase RFWD2), which contains WD40‐repeat domains, facilitates cell proliferation, transformation and cancer progression in human hepatocellular carcinoma, breast cancer and ovarian adenocarcinomas.^7^ Up‐regulation of WDR62 has been associated with centrosome amplification in human ovarian cancer and gastric cancer progression.^8^ Moreover, patients with glioblastoma (GBM) exhibiting high expression of WDR1 were shown to have poor prognosis, and signal transducer and activator of transcription (STAT) 3‐induced up‐regulation of WDR1 accelerated metastasis of triple‐negative breast cancer (TNBC).^9^ In non‐small‐cell lung cancer (NSCLC), WDR79 is frequently overexpressed and thus may serve as a potential novel diagnostic marker and therapeutic target for NSCLC.^10^ However, many mutations in the gene encoding the F‐box and the WD repeat‐containing protein 7 (FBXW7), a unique tumour suppressor that contains WD40‐repeat domains, have been detected in ovarian, breast and colorectal cancer cell lines, implicating the potential role of WD40‐repeats in the inhibition in tumorigenesis.^11^

A recent meta‐analysis of genome‐wide association studies identified two single nucleotide polymorphisms in bipolar disorder in WD‐repeat protein 41 (WDR41), a protein with six WD40‐repeat domains.^12^ WDR41 has also been implicated in dopamine signalling as a regulator of caudate volume using genome‐wide searches.^13^ Recent studies have shown that the ALS/FTLD‐associated protein, chromosome 9 open reading frame 72, in combination with the mitochondrial dynamic protein MID51 and WDR41 can act as a GDP/GTP exchange factor for the Rab family of proteins in autophagy and modulates the autophagy‐lysosomal pathway.^14^ Although previous studies have indicated that WDR5 and WDR77 form a complex with the histone methyltransferase lineage leukaemia 3 and the protein arginine methyltransferase protein arginine N‐methyltransferase 5, respectively, enhancing histone methylation,^15^, ^16^ no studies have reported the hypermethylation of the promoter region of WD‐repeat proteins in diseases. Here, we selected a variety of breast cell lines to investigate the expression of WDR41 and demonstrate the relationship between promoter methylation of WDR41 and breast cancer development.

In the present study, we demonstrated that the expression of WDR41 was lower in the TNBC cell lines than that in the tumour cell lines with low invasive capability (MCF‐7) or normal breast cell line (MCF‐10A). Furthermore, a negative correlation was detected between the WDR41 level and the pathological grade of breast cancer patients and between WDR41 and TNBC. Moreover, our results showed that aberrant methylation of WDR41 contributed to the proliferation, migration and survival of breast cancer cells via the AKT/glycogen synthase kinase‐3 beta (GSK‐3β)/β‐catenin signalling pathway. Overall, our findings suggested an inhibitory role of WDR41 in the occurrence and progression of TNBC, and provided a foundation to further explore the role of WD‐repeat proteins in cellular processes.

## 2 MATERIALS AND METHODS

### 2.1 Cell culture and cell treatment

The normal mammary epithelial cell line (MCF‐10A), oestrogen receptor (ER)‐positive breast cancer cell (MCF‐7), TNBC cell lines (MDA‐MB‐231, BT549, MDA‐MB‐468 and MDA‐MB‐453) and ER/progesterone receptor (PR)‐positive and Her2‐negative breast cancer cell lines (SKBR3 and HS578t, respectively) were purchased from American Type Culture Collection. MDA‐MB‐231 and SKBR3 cells were cultured in RPMI 1640 (SH30809.01; HyClone, USA) medium supplemented with 10% foetal bovine serum (FBS; SH30070.03; HyClone, USA). MCF‐10A and the other five cell lines were cultured in Dulbecco's modified Eagle's medium (SH30022.01; HyClone, USA) supplemented with 10% FBS. All cells were incubated in a humidified incubator containing 5% CO_2_ at 37°C. Next, 5‐aza‐dC (Sigma‐Aldrich, USA) was dissolved in dimethyl sulfoxide (DMSO) (50 mg/mL). On reaching 30%‐50% confluency, cells were exposed to 1, 5 and 10 μmol/L of 5‐aza‐dC in DMSO or DMSO alone (isometric DMSO in 10 μmol/L 5‐aza‐dC, approximately 4.5 μL) for 72 hours. After the initial 5‐aza‐dC treatment, cells were subjected to total protein and RNA extraction. For the inhibitor assay, after transfection with WDR41 small interfering RNA (siRNA; siWDR41), cells were exposed to 10 μmol/L MK‐2206 (HY‐10358; MedChemExpress, USA) for 48 hours. Following this, the total, nuclear and cytoplasmic protein fractions were extracted and analysed by Western blotting.

### 2.2 Cell transfection

The vector plasmid pEGFP‐C1 was a kind gift from Zhongxian Lv of Xiamen University, China. After obtaining the coding sequence (CDS) of WDR41 using PCR, the CDS region of WDR41 was inserted into pEGFP‐C1. For cell transfection, pEGFP‐C1 and recombinant pEGFP‐C1‐WDR41 plasmids were transfected into cells using the TurboFect transfection reagent (R0531; Thermo Fisher Scientific, USA). At 24 hours post‐transfection, cells were harvested for further investigation. For RNA interfere (RNAi)‐mediated knockdown, cells were transfected with negative control siRNA (siNC) or siWDR41 (GAUAAUAUUCUCUCAUUGA) using the TurboFect transfection reagent. At 72 hours post‐transfection, cells were used for further experiments.

### 2.3 Methyl thiazolyl tetrazolium assay

MDA‐MB‐231 cells transfected with different plasmids and siRNAs (pEGFP‐C1, pEGFP‐C1‐WDR41, siNC and siWDR41) were seeded into 96‐well plates at a density of 3 × 10^3^ cells/well and incubated for 24, 48 and 72 hours. Following incubation, 10 μL methyl thiazolyl tetrazolium (MTT) (Sigma‐Aldrich, USA) was added to the medium in each well and incubated for another 4 hours. After dissolution with DMSO, the optical density values were measured at a wavelength of 492 nm using a Multiskan plate reader (MK3; Thermo Fisher Scientific, USA).

### 2.4 Flow cytometry

For analysis of cell cycle, cells transfected with plasmids and specific siRNAs were cultured in 6‐well plates until they reached 80% confluency. Cells were then digested with trypsin without ethylene diamine tetraacetic acid and washed twice with phosphate buffered saline (PBS). After fixing with 75% ethanol at −20°C for 24 hours, the cell precipitate was stained with PI in PBS for 30 minutes in darkness. Next, all cells were analysed with a flow cytometer (Millipore, USA). For apoptosis detection, cells of all groups were seeded in 12‐well plates. After fusion growth, the cells were transferred to a centrifuge tube and washed twice with PBS. Apoptotic cells were detected with the Annexin V‐APC Apoptosis Detection Kit according to the manufacturer's instructions (KeyGEN, Nanjing, Jiangsu, China). Subsequently, the apoptosis rates were measured using a flow cytometer.

### 2.5 Cell migration assay

The migration ability of MDA‐MB‐231 cells transfected with different plasmids and siRNAs was determined by wound healing and Transwell assays. For the wound healing assay, a linear wound was created with the pipette tip (yellow pipette tip ranging from 20 to 200 μL) after cells reached a confluency of 90%‐95%, followed by incubation in serum‐free RPMI 1640 for 24 and 48 hours. Cells were then photographed at different time points, and the migration rate was calculated by measuring the wound area. The Transwell assay (PIEP12R48; Millipore, Germany) was used to evaluate the migration rate of MDA‐MB‐231 cells. For this assay, 1 × 10^5^ cells were seeded in the upper chamber containing a fibronectin‐coated polycarbonate membrane in serum‐free medium and placed in fresh medium with 10% FBS in 12‐well plates. After incubation for 12 hours, cells on the upper surface of the polycarbonate membrane were wiped off and cells that migrated to the lower surface of the membrane were stained with 0.1% crystal violet for 20 minutes at room temperature (RT). Then, the number of migrated cells was calculated using a microscope (OLYMP^®^S, IX51, Japan).

### 2.6 DNA methylation analysis

Bisulphite conversion of DNA samples was performed using the EZ DNA Methylation™ Gold Kit (ZYMO RESEARCH, USA) according to the manufacturer’ s instructions. Then, 1‐4 μL of the eluted DNA was subjected to PCR under the following conditions: 98°C for 10 minutes, 53°C for 30 minutes, 53°C for 6 minutes, followed by 8 cycles at 37°C for 30 minutes and 4°C storage. The primer sequences for MSP are shown in Table S1. The reaction products were then examined by agarose gel electrophoresis.

### 2.7 Western blotting and nuclear‐cytoplasmic protein preparation

Western blotting was performed as per a previously described method.^17^ Primary antibodies against WDR41, AKT and β‐catenin were obtained from Santa Cruz Biotechnology (USA). p‐AKT^ser473^, GSK‐3α/β and p‐GSK‐3α/β^ser21/9^ antibodies were purchased from Cell Signalling Technology (Massachusetts, USA). GAPDH (Sangon Biotech, Shanghai, China) served as the internal control. For nuclear and cytoplasmic protein preparation, proteins were extracted using the nuclear and cytoplasmic extraction kit (CW0199S; CWBiotech, Beijing, China) and subjected to Western blotting to evaluate the differentially expressed proteins in the nuclear and cytoplasmic fractions. Analysis of grey scale intensity of the immunoblots was performed using Quantity One analysis software (Bio‐Rad, California, US).

### 2.8 Quantitative real‐time PCR

Cells were subjected to total RNA extraction using the TRIzol reagent (Roche, Indianapolis, IN, USA) according to the manufacturer's instructions. After performing reverse transcription with 1 μg of RNA using the First‐Strand cDNA Synthesis kit (Transgene, China), quantitative real‐time PCR (qRT‐PCR) was performed using the SYBR Green kit (Roche, Indianapolis, IN, USA). Target gene (WDR41) expression was normalized to GAPDH expression. Relative gene levels were calculated using the 2^−∆∆Ct^ method.

### 2.9 Immunofluorescence assay

Cells were seeded on cover glasses for 24 hours, fixed with 3% paraformaldehyde for 30 minutes at 4°C and then neutralized with 50 mmol/L NH_4_Cl. After permeabilizing with 0.1% Triton‐100 for 15 minutes and washing with PBS, cells were incubated with WDR41 antibody at RT for 1 hour, followed by incubation with a secondary antibody for 1 hour. The immunolabelled cells were visualized under a Carl Zeiss LSM5 EXITER laser scanning confocal microscope (Carl Zeiss, Germany).

### 2.10 Human specimens and immunohistochemical staining

Human breast cancer tissues (grades II and III) and human breast cancer tissue chips were gifted from the First Affiliated Hospital of Xiamen University or purchased from OUTDO BIOTECH (Shanghai, China), respectively. A signed informed consent form was obtained from all subjects, and this study was approved by the ethics committee of the First Affiliated Hospital of Xiamen University. Briefly, tissue chips and tissue sections were placed in a drying oven for 1 hour at 60°C, dewaxed in xylene solution and hydrated in graded ethanol solutions. Next, antigen retrieval was performed in an autoclave for 5 minutes, followed by incubation with 0.3% H_2_O_2_ for 20 minutes. After blocking with 1% bovine serum albumin for 1 hour, the chips were incubated overnight with a primary antibody against WDR41. The next day, the tissue chip was hybridized with the secondary antibody and subjected to chromogenic reaction using DAB reagent. Comprehensive analysis included measuring the staining intensity and the number of positive cells in the tissue chip, which were analysed using the Image Pro Plus software v6.0 (negative: −; weakly positive: +, <20%; middling positive: ++, 20%‐50%; strongly positive: +++, >50%). Tumours in Table 1 are categorized as ‘low’ or ‘high’ grade based on relative WDR41 expression according to histological staining intensity (++ and +++ were defined as high, − and + were defined as low). Five high‐power fields for each sample were chosen for evaluation of relative WDR41 levels by three independent pathologists using the Image Pro Plus software v6.0 (Media Cybernetics, Inc, Maryland, USA).

**TABLE 1:** The percentage of patients with low WDR41 expression in all breast cancer patients, including TNBC

Patients (14)	Numbers	Rates
ER‐, HER2‐, PR‐	8	57.14% (8/14)
WDR41 low expression	11	78.57% (11/14)
WDR41 low expression in TNBC	6	75% (6/8)

Note

There were 14 breast cancer patients, of which 8 (57.14%) were TNBC patients. Among the 14 patients, 11 (78.57%) had low WDR41 expression levels. Of note, 75% (6/8) of the TNBC patients showed low levels of WDR41.

Abbreviations: ER, oestrogen receptor; PR, progesterone receptor; TNBC, triple‐negative breast cancer; WDR41, WD‐repeat protein 41.

### 2.11 Xenograft experiment

Male BALB/c nude mice (5‐6 weeks of age, 16‐18 g) were obtained from the Laboratory Animal Centre of Xiamen University, China. Mice were housed in a specific pathogen‐free room with controlled temperature (20 ± 2°C) and humidity. All mice were exposed to a 12‐hours light‐dark cycle with free access to standard rodent chow and water. After a week of adjustable feeding, mice were randomly assigned to two groups (n = 8/each group): mice inoculated subcutaneously into the right side of the axillary area with 2 × 10^6^ MDA‐MB‐231 cells (100 μL) transfected with pEGFP‐C1 or pEGFP‐C1‐WDR41. At day 7 post‐inoculation, tumour size measurements in the two groups were performed using vernier callipers every two days. At day 19 post‐injection, mice were killed by cervical dislocation and imaged with a camera. Next, tumours in each mouse were stripped, photographed and harvested for subsequent experiments. Tumour volume was calculated using the formula: 0.5 × length × width^2^. All animal studies were approved by the Animal Welfare Committee of Research Organization, Xiamen University (approval number: XMULAC201511).

### 2.12 Statistical analyses

All Western blotting and qRT‐PCR experiments were independently performed at least three times using independent samples. For the analysis of staining intensity, the number of migrated cells was measured on the basis of at least five horizons in different samples. Quantitative analysis of Western blotting data, gene expression and tumour xenograft‐related analyses was performed using the GraphPad Prism 5.0 software^18^ with a two‐tailed Student's *t* test. MTT, wound healing and apoptosis assay data were analysed by two‐way analysis of variance (ANOVA) using GraphPad Prism. Statistical analysis of clinical correlation was performed by the Cochran‐Mantel‐Haenszel and chi‐squared tests. Values have been presented as mean ± standard error of mean. *P* < .05 was considered to be statistically significant.

## 3 RESULTS

### 3.1 WDR41 is expressed in human TNBC

**FIGURE 1:**
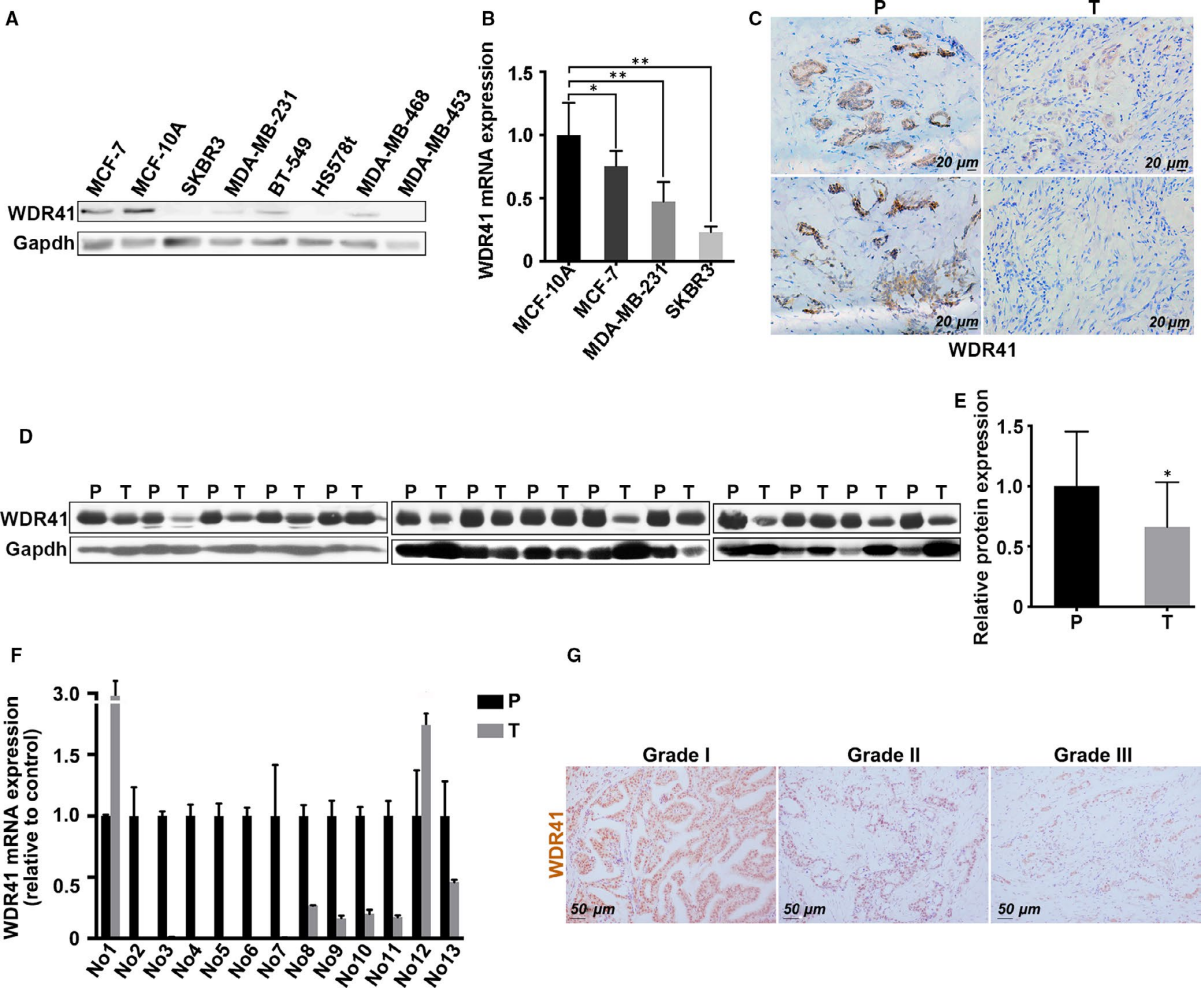
Expression pattern of WDR41 in human breast cancer cells and tissues (A) Expression of WDR41 in breast cancer cell lines was evaluated by Western blotting. MDA‐MB‐231, BT549, MDA‐MB‐468 and MDA‐MB‐453 were the TNBC cell lines; MCF‐10A and MCF‐7 were the normal breast cell line and low invasive capability cells, respectively. The SKBR3 cell line was ER‐ and PR‐negative, but Her2‐positive. B, The mRNA level of WDR41 was determined by qRT‐PCR in normal mammary epithelial cells (MCF‐10A) and breast tumour cells (MCF‐7, MDA‐MB‐231 and SKBR3). C, Immunohistochemical staining of WDR41 in human breast cancer samples and corresponding adjacent tissues. Scale bar: 20 μm. D, Protein expression of WDR41 in human breast cancer tissues and paired para‐carcinoma tissues was measured by Western blotting. E, Relative protein expression of WBP2 in panel D. F, The mRNA level of WDR41 in human breast cancer tissues and its paired para‐carcinoma tissues was measured by qRT‐PCR. G, Immunohistochemical staining with WDR41‐specific antibody in different pathological grades of human breast cancer tissue chips. Scale bar: 50 μm. P, para‐carcinoma, T, tumour. *P < .05, **P < .01. ER, oestrogen receptor; PR, progesterone receptor; qRT‐PCR, quantitative real‐time PCR; TNBC, triple‐negative breast cancer; WDR41, WD‐repeat protein 41

To obtain insights into the cellular function of WDR41, we evaluated its distribution within several cell types. Immunostaining results indicated that WDR41 displayed a diffuse localization pattern in HeLa, MDA‐MB‐231 and MCF‐7 cells, suggesting that it has a wide range of functions in cellular regulation (Figure S1A). Based on a previous study on the role of WD‐repeat proteins in tumorigenesis, we first examined the expression pattern of WDR41 in breast cancer cell lines. As shown in Figure 1A, WDR41 levels were very low in TNBC cells, such as MDA‐MB‐231, BT549, MDA‐MB‐468 and MDA‐MB‐453 cells compared to that in normal breast cells (MCF‐10A). The cell line SKBR3, which is ER‐ and PR‐negative but Her2‐positive, also presented low WDR41 levels. Moreover, we determined the mRNA levels of *WDR41* in normal mammary epithelial cells (MCF‐10A) and breast cancer cells (MCF‐7, MDA‐MB‐231 and SKBR3 cells). qRT‐PCR results revealed that the mRNA expression of *WDR41* was notably decreased in breast cancer cells compared to that in normal MCF‐10A cells, indicating lower WDR41 levels in cell lines with a high invasive capability (MDA‐MB‐231: a 50% fall, *P* = .003; SKBR3: a 75% fall, *P* = .0001) than those in MCF‐7 cells with a lower invasive capability (a 30% fall, *P* = .0473) (Figure 1B).

Given the down‐regulated expression of WDR41 in breast cancer cells, we analysed its expression pattern in human breast cancer samples (grades II and III) to explore the relationship between WDR41 and breast cancer development. Immunohistochemical staining results showed a higher expression of WDR41 in para‐carcinoma tissues than in paired tumour tissues (Figure 1C). After assessing the protein level of WDR41 in 14 pairs of human breast cancer tissues and their corresponding normal breast tissues, we found that WDR41 level was reduced in approximately 78.6% (11/14) of the tumour samples compared to that in paired normal breast tissue samples (Figure 1D,E). Owing to limited number of clinical cases, we only obtained 13 pairs of breast tissues to verify the mRNA expression level of *WDR41*. Among these clinical tissue samples, approximately 85% of the tumour tissues (11/13) presented extremely low WDR41 levels as compared with their matching normal tissues (Figure 1F). To determine the correlation between WDR41 levels and TNBC in human specimens, we further analysed the expression of ER, PR and Her2 in the human breast cancer specimens. Combined with the matched data provided by the hospital, we found that 8 out of the 14 breast cancer specimens showed low or no expression of ER, PR and Her2. In addition, 75% of patients with TNBC (6/8) presented low protein and mRNA levels of WDR41 (Figure 1D‐F and Table 1), implying that 6 out of 11 (54.5%) patients expressing low WDR41 levels belonged to the TNBC group. To ascertain the direct relationship between WDR41 expression and breast cancer, we evaluated the expression of WDR41 in human breast cancer chips, which contained 61 clinical stage samples, as well as the status of breast tumour tissues using immunohistochemical staining. A total of 1 male and 60 female breast cancer patients were included in this tissue microarray, and 47 patients were older than 50 years of age. Among these patients, 3 and 59 patients showed lymph node metastasis and invasive pathological type, respectively. In addition, 40 patients had TNM grades I‐II and 21 had grades III‐IV tumours (Table 2). Based on the WDR41 status along with immunohistochemical staining results, all samples were divided into two groups. The data showed that 35 out of 61 (57.3%) patients presented low expression of WDR41, and 26 out of 61 (42.7%) patients displayed a strong positive staining for WDR41 (Table 2). Correlation analysis indicated that WDR41 status had no correlation with the clinicopathological characteristics of patients, including sex, age, lymph node metastasis, TNM grade and pathological type. However, patients with advanced grade (grade 2: G2 and grade 3: G3) always showed low WDR41 expression compared to patients with early grade tumours (G1) (*P* = .0002) (Figure 1G and Table 2). After integrating gene chip information, we observed that 22 out of 61 patients showed negative staining for ER, PR and Her2, of which 19 specimens (54.3%, 19/35) presented low WDR41 expression and 3 samples (11.5%, 3/26) presented high WDR41 expression, thus implying that low levels of WDR41 were partially correlated with TNBC (*P* = .0369) (Table 2). Taken together, these findings demonstrate that WDR41 expression is decreased in human breast cancer cells and breast cancer specimens. Of note, low levels of WDR41 may most likely be observed in TNBC specimens.

**TABLE 2:** Chi‐squared analysis of contingency tables between WDR41 expression and clinicopathological characteristics of patients with breast cancer

Characteristics	Relative WDR41 expression		*P* value
	Low (n = 35)	High (n = 26)	
Gender			.4262
Male	0	1	
Female	35	25	
Age			.5530
≤50	7	7	
>50	28	19	
Tumour grade			.0002**
G1	1	12	
G2	19	8	
G3	15	6	
ER‐, HER2‐, PR‐			
G1	0	0	.0369*
G2	6	2	
G3	13	1	
Lymph node metastasis			1.0000
No	33	25	
Yes	2	1	
TNM grade			.7710
I + II	20	20	
III + IV	15	6	
Tumour diameter (cm)			.7309
≤5	30	21	
>5	5	5	
Pathological type			1.0000
Non‐invasive	1	1	
Invasive	34	25	

Abbreviation: ER, oestrogen receptor; PR, progesterone receptor; WDR41, WD‐repeat protein 41.

a * *P* < .05, ** *P* < .01, was considered statistically significant.

### 3.2 WDR41 promoter region is highly methylated in MDA‐MB‐231 cells

**FIGURE 2:**
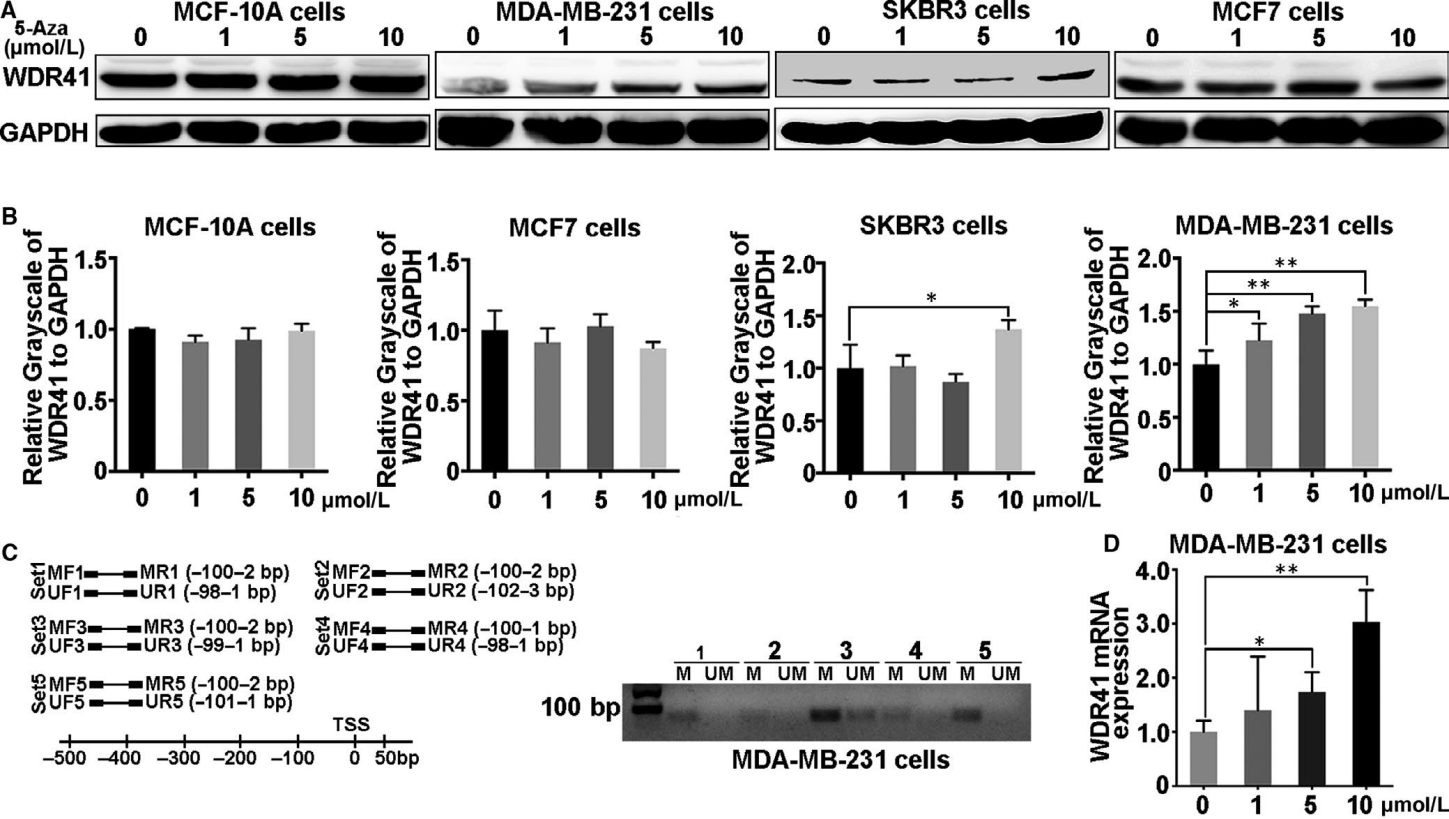
Methylation level of the WDR41 promoter region in MDA‐MB‐231 cells. A, Protein expression of WDR41 was evaluated in MCF‐10A, MDA‐MB‐231, SKBR3 and MCF‐7 cells after treatment with different doses of 5‐aza‐dC. B, Relative quantification of WDR41 protein expression in MCF‐10A, MDA‐MB‐231, SKBR3 and MCF‐7 cells after stimulation with different doses of 5‐aza‐dC. C, Methylation‐specific PCR was performed to measure the methylation level of WDR41 in MDA‐MB‐231 cells. D, The mRNA level of WDR41 in MDA‐MB‐231 cells after pre‐treatment with increasing doses of 5‐aza‐dC. M, methylation primer, UM, unmethylated primer. *P < .05, **P < .01. WDR41, WD‐repeat protein 41

Gene expression is regulated by various factors, including microRNAs, transcription factors and epigenetic changes. Owing to WDR41 hypermethylation in leukoaraiosis, observed through DNA methylation chip (unpublished data), we hypothesized that WDR41 expression was potentially governed by DNA methylation in breast cancer as well. First, we determined the protein level of WDR41 in breast cancer cells using 5‐aza‐dC, an inhibitor of DNA methylation, to verify our assumption. An increase in 5‐aza‐dC dosage (1, 5 and 10 μmol/L) did not affect the expression of WDR41 in MCF‐10A and MCF‐7 cells, and only approximately 30% WDR41‐up‐regulation was observed in SKBR3 cells at a dosage of 10 μmol/L (*P* = .0283) (Figure 2A,B). However, a corresponding dose‐response was also observed at the protein level in MDA‐MB‐231 cells, wherein WDR41 levels increased by 25% (*P* = .0326), 50 (*P* = 0.0046) and 60% (*P* = .0026) after stimulation with 1, 5 and 10 μmol/L 5‐aza‐dC, respectively (Figure 2A,B). Based on this result, we evaluated the methylation level of the WDR41 promoter region in MDA‐MB‐231 cells by MSP with five pairs of primers designed using the MethPrimer method (Table S1). MSP data indicated that hypermethylated WDR41 was found in the TNBC cell line MDA‐MB‐231 (Figure 2C). After treatment with different concentrations of 5‐aza‐dC, the mRNA level of *WDR41* in MDA‐MB‐231 cells significantly increased by 65% (*P* = .0103) and 2 times (*P* < .0001) at doses of 5 and 10 μmol/L 5‐aza‐dC, respectively (Figure 2D). Therefore, these results indicate that WDR41 expression in MDA‐MB‐231 cells is potentially governed by DNA methylation.

### 3.3 WDR41 affects the proliferation and migration of TNBC cells

**FIGURE 3:**
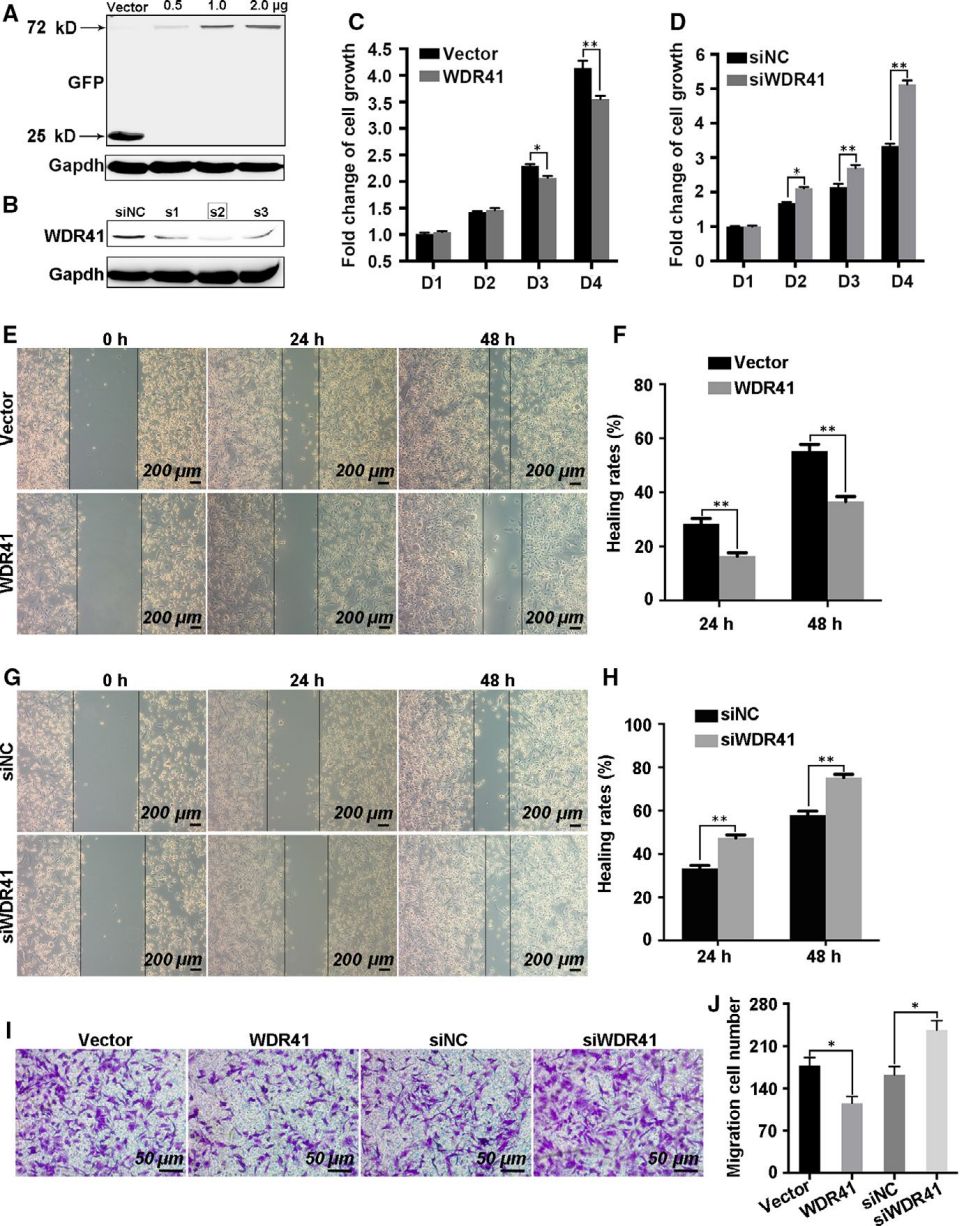
Effect of WDR41 on the growth and metastatic ability of MDA‐MB‐231 cells (A and B) Validation of WDR41 or WDR41‐siRNA transfection efficiency in MDA‐MB‐231 cells. Here, 0.5. 1.0 and 2.0 indicate the mass of the plasmid, and S1, S2 and S3 indicate three different WDR‐siRNA oligos. C and D, MTT assay was performed to evaluate cell growth in the four different groups. Scale bar: 200 μm. E and F, Images of wound healing and healing rates in the vector and WDR41 groups after seeding for 24 and 48 h. Scale bar: 200 μm. G and H, Images of wound healing and healing rates in siNC and siWDR41 groups after seeding for 24 and 48 h. I, The Transwell assay results have been presented here for the four different groups. Scale bar: 50 μm. J, The average number of migrated cells in 5 high‐power fields in the vector, WDR41, siNC, and siWDR41 groups. Vector, EGFP group (control); WDR41, EGFP‐WDR41 group; siNC, negative control group; siWDR41, WDR41‐siRNA group. *P < .05, **P < .01. MTT, methyl thiazolyl tetrazolium; WDR41, WD‐repeat protein 41

Aberrant methylation of WDR41 in MDA‐MB‐231 cells indicated that it might have acted as a tumour suppressor gene in the progression of TNBC. In order to assess the cellular functions of WDR41, cell proliferation and migration assays were performed in MDA‐MB‐231 cells transfected with exogenetic WDR41 and siRNAs targeting WDR41. Based on the knockdown efficiency, siRNA‐2 was used for further experiments (Figure 3A). Results showed a significant suppression of cell viability in the WDR41 overexpressing group compared with that in the control vector group (cells transfected with the vector control plasmid) on days 3 (*P* = .0103) and 4 (*P* < .0001) (Figure 3A,C), while RNAi‐mediated knockdown of WDR41 resulted in 30% (*P* = .023), 35% (*P* < .0001) and 57% (*P* < .0001) up‐regulation in cell viability in the WDR41 group compared to that in the vector group on days 2 to 4, respectively (Figure 3B,D). The migration assay results showed that the wound area and healing rate significantly decreased by 33% at 24 hours (*P* = .0044) and 35% at 48 hours (*P* = .0002) post‐wounding in the vector group compared to that in the WDR41 group (Figure 3E,F). Moreover, WDR41 depletion increased by 32% at 24 hours (*P* < .0001) and 36% at 48 hours (*P* < .0001) in wound healing rate as compared to that in the control cells (Figure 3G,H). Additionally, the Transwell assay results showed a 35% reduction (*P* < .0001) in migration rate following overexpression of WDR41 and in contrast to a 46% increase (*P* < .0001) in migration rate observed in WDR41‐depleted cells, as compared with the corresponding control cells (Figure 3I,J). We also employed BT549 cells to clarify the cellular functions of WDR41 in TNBC cells. Consistently, forced expression of WDR41 robustly inhibited, while ablation of WDR41 promoted BT549 cell viability and migration (*P* < .01) (Figure S2A‐D). Thus, these results indicate that WDR41 has potential anti‐neoplastic effects on TNBC cells.

### 3.4 WDR41 induces cell cycle arrest and apoptosis in MDA‐MB‐231 cells

**FIGURE 4:**
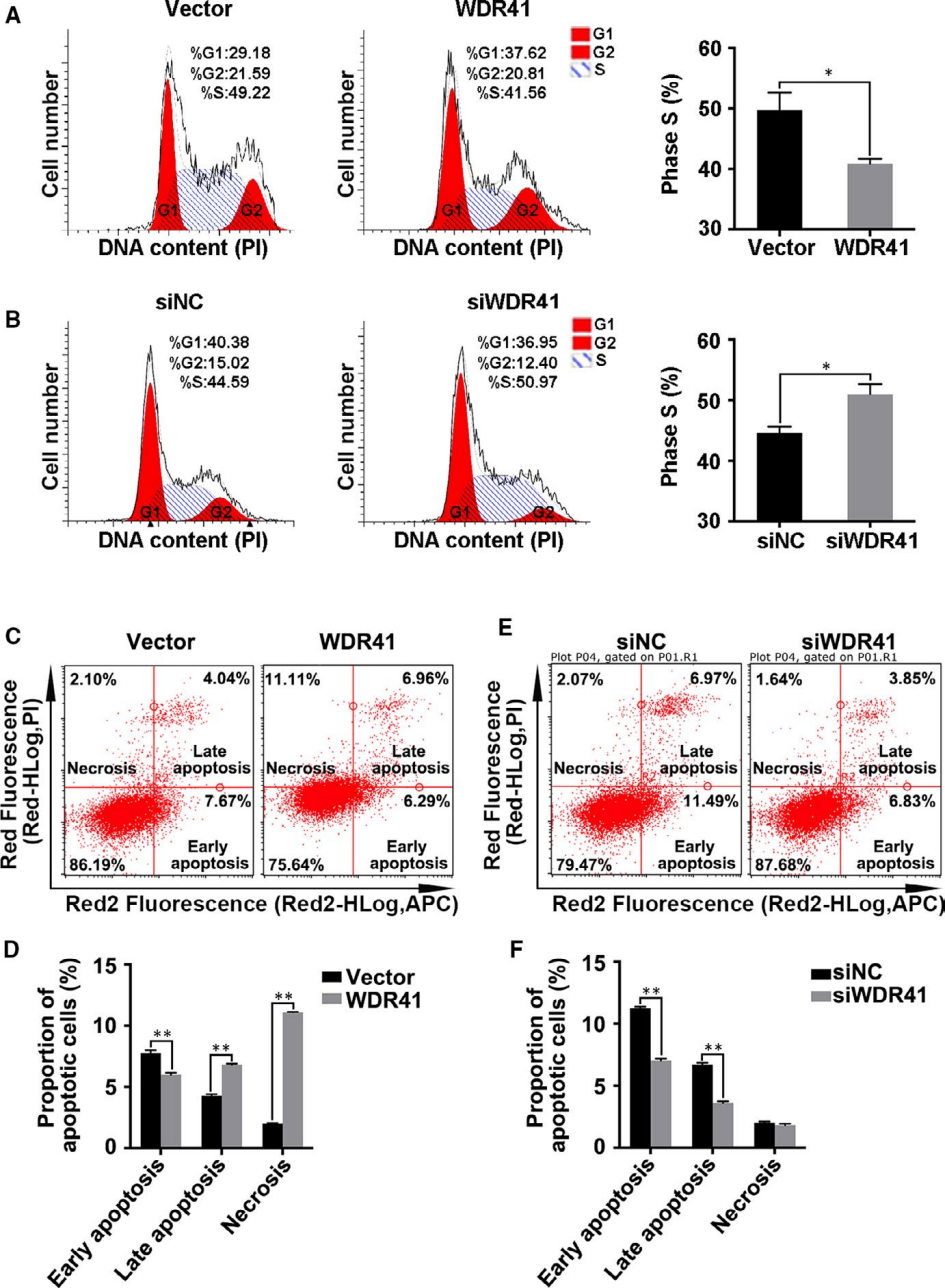
The impact of WDR41 on cell cycle and apoptosis of MDA‐MB‐231 cells. Cell cycle progression was analysed in MDA‐MB‐231 cells transfected with vector and WDR41 plasmids (A) or negative control and WDR41‐siRNA (B). C, The effect of WDR41‐up‐regulation on cell apoptosis was analysed by flow cytometry. D, The proportion of apoptotic cells was measured in the vector and WDR41 groups. E, The effect of WDR41‐down‐regulation on cell apoptosis was analysed by flow cytometry. F, The proportion of apoptotic cells was measured in the siNC and siWDR41 groups. Vector, EGFP group (control); WDR41, EGFP‐WDR41 group; siNC, negative control group; siWDR41, WDR41‐siRNA group. *P < .05, **P < .01. WDR41, WD‐repeat protein 41

Next, we performed flow cytometry analysis to determine the impact of WDR41 on cell cycle and programmed cell death. Results showed that forced expression of WDR41 in MDA‐MB‐231 cells decreased the number of S phase cells by 17% (*P* = .0166) (Figure 4A). However, WDR41 ablation increased the proportion of S phase cells by 15% (*P* = .0215) compared to that in siNC group (Figure 4B). Furthermore, results of the apoptosis assay revealed a significant increase in the proportion of late apoptotic cells by 60% (*P* < .0001) and necrotic cells by 4.6 times (*P* < .0001) in WDR41‐overexpressing cells, with a 23% increase (*P* < .0001) in the proportion of early apoptotic cells (Figure 4C,D). Conversely, knockdown of WDR41 markedly reduced the proportion of early apoptotic and late apoptotic cells by 37% (*P* < .0001) and 46% (*P* < .0001), respectively (Figure 4E,F). Accordingly, our findings demonstrated that WDR41 moderately abolished the DNA synthesis, resulting in cell cycle arrest and a subsequent increase in apoptosis.

### 3.5 Overexpression of WDR41 represses tumour formation in MDA‐MB‐231 cells

**FIGURE 5:**
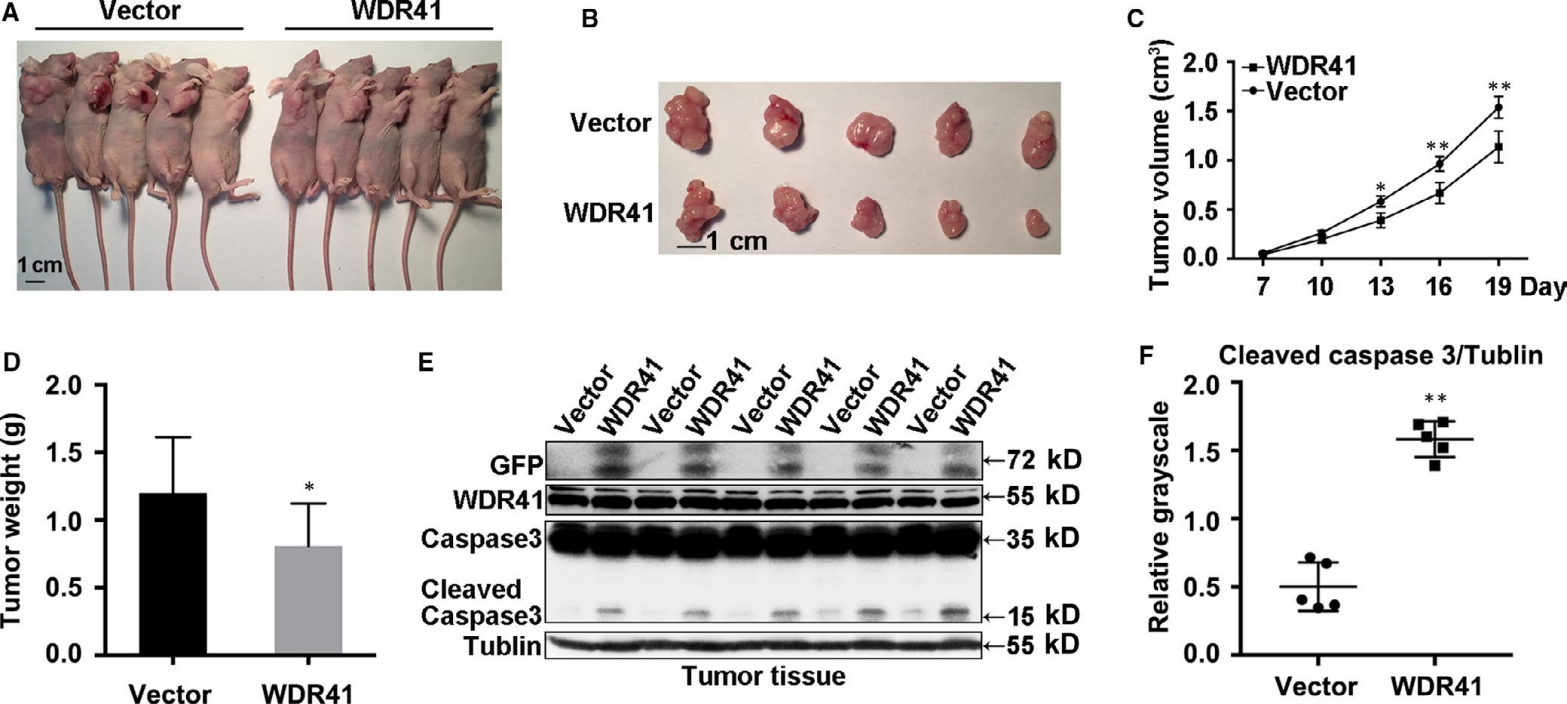
Effect of WDR41 on the tumorigenic ability of MDA‐MB‐231 cells (A) Images of a xenograft tumour model of vector and WDR41 groups. n = 5 per group. B, Images of the size of tumours in the vector and WDR41 groups. C, Tumour growth curves of the vector and WDR41 groups. D, Tumour weights of the two groups. E, Protein levels of WDR41, caspase 3 and cleaved caspase 3 in the mice tumours. Tubulin served as the internal control. F, Relative quantification of the cleaved caspase 3 in panel E. Vector, EGFP group (control); WDR41, EGFP‐WDR41 group. Scale bar: 1 cm. *P < .05, **P < .01. WDR41, WD‐repeat protein 41

Since WDR41 seemed to act as a tumour suppressor gene in breast tumorigenesis, we sought to identify whether WDR41 also demonstrated similar biological properties in vivo. Using MDA‐MB‐231 cells transfected with exogenous WDR41 and blank vector control, we established a tumour xenograft model in BALB/c nude mice (n = 8/group). As shown in Figure 5A, up‐regulation of WDR41 significantly attenuated the tumorigenic ability of MDA‐MB‐231 cells. We also observed a significant reduction in tumour size from the tumour images obtained (Figure 5B). Combined with the tumour growth curves, results showed that the tumour volume was decreased by 33% (*P* = .0470), 31% (*P* = .009) and 26% (*P* = .0021) reduction on days 13, 16 and 19, respectively, in mice inoculated with WDR41‐overexpressing MDA‐MB‐231 cells compared to that in mice inoculated with control cells (Figure 5C). Additionally, compared to that in the control group, tumour weight in the WDR41‐overexpressing group was markedly reduced by approximately 33% (*P* = .0462; Figure 5D). Furthermore, the decreased tumour volume in the WDR41‐overexpressing group was not found to be associated with overall bodyweight, which was not significantly different between the WDR41‐overexpressing and control groups (Figure S1B). Based on the apoptosis promotion function of WDR41 in vitro, we evaluated the levels of the apoptotic marker cleaved caspase 3 in tumours. Compared to that in the vector group, the level of cleaved caspase 3 was increased by 3‐fold (*P* < .0001) in the WDR41‐overexpressing group (Figure 5E,F). Overall, our results indicate that overexpression of WDR41 inhibits the in vivo tumorigenic ability of MDA‐MB‐231 cells.

### 3.6 The GSK‐3β/β‐catenin pathway is inhibited by up‐regulation of WDR41

**FIGURE 6:**
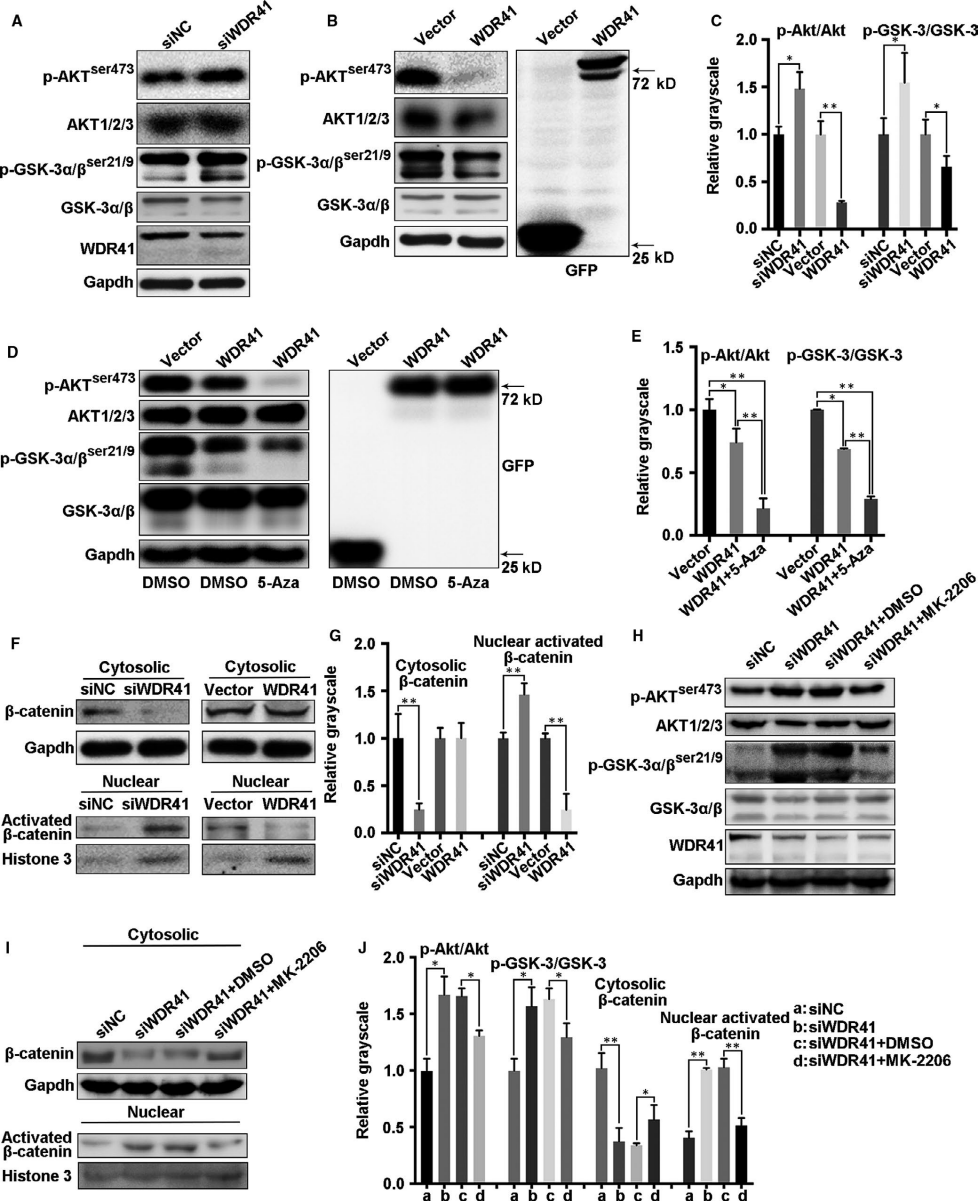
Regulatory mechanism of WDR41‐mediated tumour inhibition. A, The activation of AKT and GSK‐3α/β was detected by Western blotting after knockdown of WDR41 in MDA‐MB‐231 cells. B, The activation of AKT and GSK‐3α/β was detected by Western blotting after up‐regulation of WDR41 in MDA‐MB‐231 cells. C, Relative quantification of phosphorylated AKT and GSK‐3α/β in the siNC, siWDR41, vector and WDR41 groups. Protein levels of phosphorylated AKT and GSK‐3α/β (D) and their relative quantification (E) in DMSO‐treated vector and WDR41 groups and 5‐aza‐dC‐treated WDR41 group. F, The distribution of β‐catenin in the cytoplasm and nucleus of MDA‐MB‐231 cells was measured by extracting the nuclear and cytoplasmic protein fractions, followed by Western blotting. G, Relative quantification of cytoplasmic β‐catenin and activated nuclear β‐catenin. H, After transfection with siWDR41, the cells were exposed to DMSO or MK‐2206. The activation of AKT and GSK‐3α/β was detected by Western blotting in MDA‐MB‐231 cells. I, After treatment with siWDR41 and MK‐2206, the nuclear and cytoplasmic protein fractions were extracted and the distribution of β‐catenin was measured in MDA‐MB‐231 cells by Western blotting. J, Relative quantification of the phosphorylated AKT and GSK‐3α/β in panel H and nuclear and cytoplasmic β‐catenin in panel I. *P < .05, **P < .01. DMSO, dimethyl sulfoxide; WDR41, WD‐repeat protein 41

Previous studies have reported that the Wnt/β‐catenin signalling is involved in the regulation of WD repeat‐containing protein expression, with the likelihood of a possible feedback mechanism in some cases.^19^ Consequently, we verified the correlation between the Wnt/β‐catenin pathway and WDR41 in TNBC cells. As expected, ablation of WDR41 promoted the activation of GSK‐3α/β^ser21/9^ (*P* = .0121) and AKT^ser473^ (*P* = .0468) by approximately 1.5‐fold, both of which are key determinants of the Wnt/β‐catenin signalling pathway (Figure 6A,C), whereas overexpression of WDR41 restrained the phosphorylation of AKT^ser473^ and GSK‐3α/β^ser21/9^ by 75% (*P* = .0009) and 30% (*P* = .0376), respectively (Figure 6B,C). Of note, 5‐aza‐dC treatment had amplification effects on WDR41‐up‐regulation and induced the inactivation of phosphorylated AKT^ser473^ (down‐regulation by 71% vs. 26%) and GSK‐3α/β^ser21/9^ (down‐regulation by 59% vs. 31%) compared to that by DMSO treatment, implying that 5‐aza‐dC‐mediated restoration of WDR41 level could disturb the GSK‐3α/β and AKT signalling (Figure 6D,E). Furthermore, WDR41 depletion resulted in an increase in the nuclear β‐catenin level by 1.5‐fold (*P* = .0038) and decrease in cytoplasmic β‐catenin level by 75% (*P* = .0080) compared to the corresponding location of β‐catenin in control MDA‐MB‐231 cells; however, there was a 75% reduction (*P* = .0017) in the nuclear activation of β‐catenin in WDR41‐overexpressing cells compared with that in the control cells (Figure 6F,G). Next, we used the AKT inhibitor (MK‐2206) to provide mechanistic insights into the role of WDR41 in regulating the GSK‐3β/β‐catenin signalling pathway in MDA‐MB‐231 cells. The results indicated that the activities of p‐AKT^ser473^ and p‐GSK‐3α/β^ser21/9^ in the cytoplasm (Figure 6H,J, the second column vs. the first column) and activated β‐catenin in the nucleus (Figure 6I,J, the second column vs. the first column) were robustly enhanced by approximately 1.7‐, 1.6‐ and 2.5‐fold, respectively, while the cytoplasmic protein level of β‐catenin was evidently reduced by 60% in the absence of WDR41. However, MK‐2206 administration significantly inhibited the siWDR41‐mediated elevation of phosphorylated AKT^ser473^ and GSK‐3α/β^ser21/9^ by about 25%, with a subsequent reduction of the nuclear β‐catenin level by 50% and restoration of the cytoplasmic β‐catenin level (Figure 6H‐J). Thus, our findings indicate that WDR41 negatively affects the GSK‐3β/β‐catenin pathway in an AKT‐dependent manner in MDA‐MB‐231 cells.

## 4 DISCUSSION

Breast cancer is one of the most common invasive cancers and the leading cause of cancer‐related deaths in women globally.^20^ The prognosis of breast cancer depends on various factors, ranging from age and lifestyle to the stage of cancer. Currently, a combination of surgery and chemotherapy or radiation therapy is the most common treatment for breast cancer; however, poor prognosis and relapse still occur frequently.^21^ Although advanced therapeutic strategies, such as targeted cancer therapy and combination therapy, are constantly emerging, they are associated with several limitations.^22^, ^23^, ^24^, ^25^ Therefore, there is an urgent need to identify effective therapeutic targets to combat breast cancer.

Similar to other types of cancer, human breast cancer also exhibits a high number of epigenetic alterations in the genome.^26^ DNA methylation alterations associated with cancer‐related genes have become more pronounced in the TNBC subtype.^27^ In the present study, we found that WDR41 was weakly expressed in TNBC cells and tissues. Hypermethylated WDR41 was detected in MDA‐MB‐231 cells, and DNA demethylation notably rescued the low levels of WDR41 in TNBC cells. These data implied that down‐regulation of WDR41 in TNBC cells might be partly due to the aberrant methylation of WDR41. Many human ubiquitin‐specific proteases (USPs), including USP7, USP12 and USP46, interact with the WD40‐repeat proteins to mediate diverse cellular processes.^28^ As a biomarker of breast cancer stem cells, USP44 interacts directly with the WD40 motif‐containing proteins, such as the N‐CoR component, TBL1XR1, which is highly expressed in breast cancer cells.^29^, ^30^ Of note, USP44 deubiquitinates histone H2B both in vivo and in vitro*,* which contributes to N‐CoR (USP44 is a part of the N‐CoR complex)‐mediated repression of target genes.^31^, ^32^ Monoubiquitinated H2B is required in human cells for histone H3 methylation on lysine 4 (H3K4) and lysine 79 (H3K79).^33^, ^34^ As a WD40‐repeat protein, down‐regulation and aberrant methylation of WDR41 in TNBC cells may possibly be involved in the USP44‐mediated deubiquitination of H2B.

Extensive studies have claimed that the WD40‐repeat proteins generally function as platforms of protein‐protein interactions and influence cell proliferation, invasion and survival by regulating DNA production and cell cycle progression.^35^ The MYC‐WDR5 nexus has been shown to promote induced pluripotent stem cell generation and drive oncogenesis, and WDR5, as a key determinant of MYC recruitment to chromatin, may be an effective target for developing anti‐tumour medicaments against MYC‐driven tumours.^36^, ^37^ Furthermore, microRNA‐92a was shown to directly bind to FBXW7 and, in turn, repress the expression of FBXW7, thus triggering the tumour growth in osteosarcoma.^38^ In addition, the interaction between the beta‐transducin repeat‐containing E3 ubiquitin protein ligase (βTrCP) and the SMAD‐specific E3 ubiquitin protein ligase 1 through the WD40‐repeat domains [7 × tryptophan (W) aspartic acid (D)] of βTrCP is relatively resistant to the proliferative capacity of liver cancer cells and may be useful for oncotherapy in patients with liver cancer.^39^ Here, our findings demonstrated that WDR41 affected the tumorigenesis of TNBC cells by regulating cell proliferation, migration, apoptosis and tumour growth in vivo and that WDR41 may act as a tumour suppressor of TNBC cells. Interestingly, proteins containing WD40 domains have been shown to be involved in cell cycle regulation, chromatin dynamics and DNA damage response, which are essential intracellular events for cell growth and apoptosis.^40^, ^41^ Besides, WDR5 affects cell cycle progression, histone methylation and DNA damage by regulating ubiquitination signals.^42^, ^43^, ^44^ A previous study reported that exogenous WDR41 mediated cell cycle arrest by enhancing the proportion of cells in the G0/G1 phase and restraining DNA synthesis in the S phase. Hence, we hypothesized that WDR41 overexpression‐induced inhibition of cell growth and promotion of apoptosis may be due to the activation of DNA damage, which may be mediated through ubiquitination‐associated signal transduction.

Multiple signalling pathways are involved in WD40‐repeat protein‐mediated promotion or inhibition of tumour development. WDR77 can directly interact with the transforming growth factor β‐stimulated clone 22 domain family member 2, which has been implicated as a tumour‐associated gene that exhibits diverse physiological functions, and other functions in gene transcription, cellular metabolism, cell cycle regulation and tumorigenesis.^45^ FBXW7, as a WD40 repeat‐containing protein, targets numerous oncoproteins, including mammalian target of rapamycin, c‐myc, cyclin E, c‐Jun and steroid receptor coactivator‐3 for ubiquitination‐mediated degradation and inhibition of tumour growth.^46^, ^47^, ^48^, ^49^, ^50^ FBXW7 also influences the proliferation and survival of pancreatic cancer cells through the Ras/Raf/MEK/ERK signalling cascade.^51^ In this study, forced expression of WDR41 inhibited AKT/GSK‐3β phosphorylation and activated the nuclear β‐catenin accumulation in MDA‐MB‐231 cells. Wnt/β‐catenin signalling was shown to have an inhibitory effect on microRNA‐770 expression and further suppressed FBXW7 expression, resulting in the enhancement of hepatocellular carcinoma.^52^ As a tumour suppressor in GBM, F‐box protein 16 proteasomally degrades nuclear β‐catenin, and subsequently βTrCP, in a GSK‐3β‐independent manner in GBM cells, leading to inhibition of proliferation.^53^ Here, WDR41 negatively regulated oncogenesis in TNBC cells partly by inactivating the GSK‐3β/β‐catenin pathway. Additionally, inhibition of AKT activity notably abolished the effect of WDR41‐up‐regulation on the GSK‐3β/β‐catenin pathway. AKT plays a key role in TNBC progression via modulating WDR41‐mediated inactivation of GSK‐3β/β‐catenin signalling. Of note, this inhibitory effect of WDR41 on AKT/GSK‐3β was enhanced by the addition of a methylation inhibitor. Since WDR41 methylation has been hypothesized to be associated with ubiquitylation events, there may be some relationship between the AKT/GSK‐3β/β‐catenin signalling and the ubiquitylation reaction that was linked by WDR41. Furthermore, it has been reported that DNA methyltransferase inhibitors can preferentially target hypermethylated tumour‐related genes, exerting anti‐tumour activity in TNBC cells, and may be used for epigenetic therapies for TNBCYYY.^54^, ^55^ Hence, modifications in WDR41 methylation may potentially facilitate the treatment of TNBC.

**FIGURE 7:**
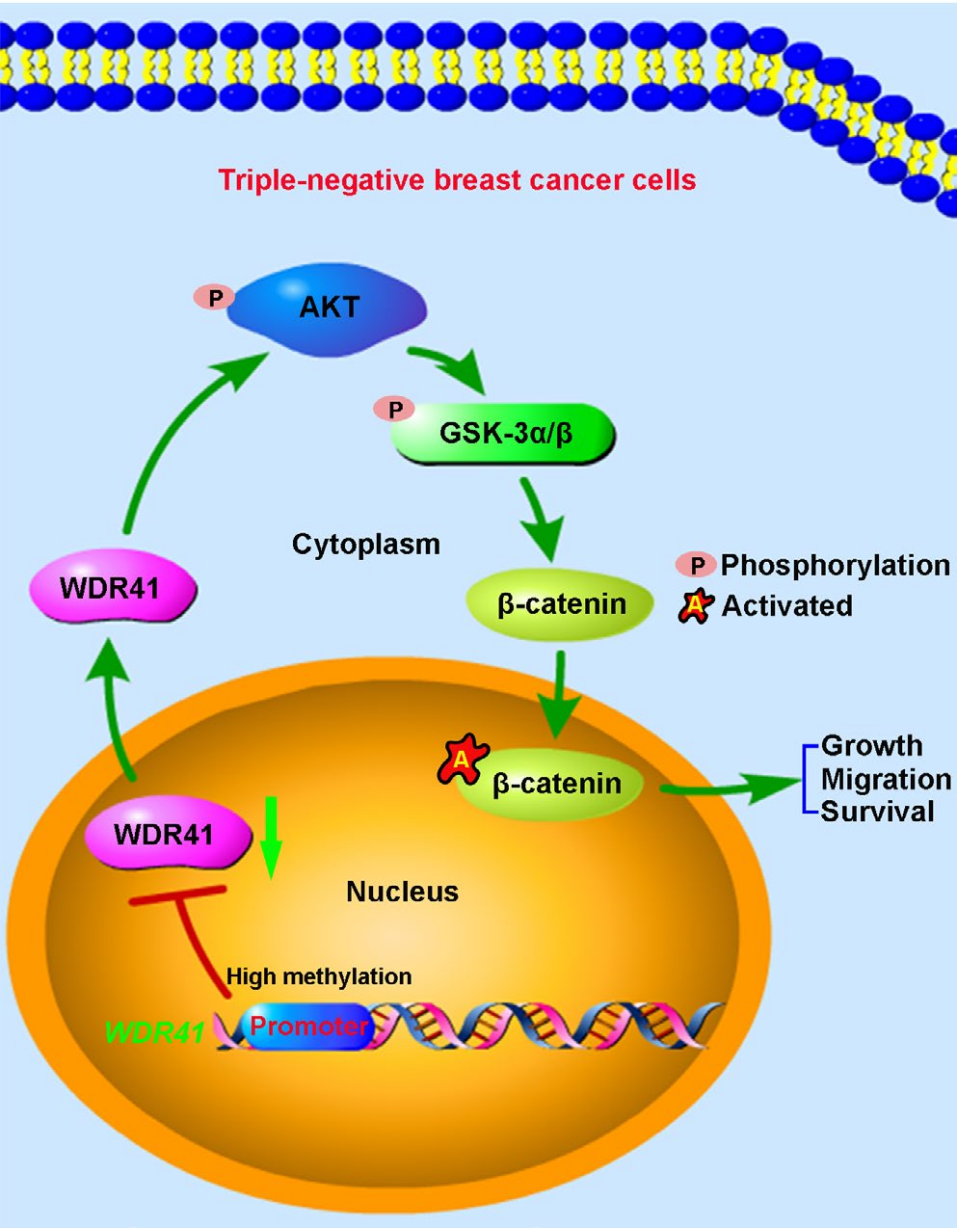
The proposed model of WDR41‐mediated cellular processes in TNBC cells. In the tumour microenvironment, high methylation across the WDR41 promoter region mediates the down‐regulation of WDR41, resulting in an excessive activation of the AKT/GSK‐3β signalling pathway and subsequent activation of the nuclear β‐catenin, leading to promotion of tumour progression in TNBC. TNBC, triple‐negative breast cancer; WDR41, WD‐repeat protein 41

In conclusion, our results showed that hypermethylation of WDR41 was detected in MDA‐MB‐231 cells. Further, WDR41 demethylation or up‐regulation suppressed the proliferative, migratory and tumour formation abilities of MDA‐MB‐231 cells by negatively regulating the AKT/GSK‐3β/β‐catenin signalling pathway, and this effect was attenuated by knockdown of WDR41 (Figure 7). Collectively, our findings may have a critical impact on the development of therapeutic strategies for TNBC management and provide new insights into the role of WD40‐repeat proteins and DNA methylation in TNBC.

## CONFLICT OF INTERESTS

No competing interests declared.

## AUTHORS' CONTRIBUTIONS

SC and ZM conceived the idea. SC drafted the manuscript. WH and WD designed and performed the experiments. SC and WD analysed the data and designed the figures. LL contributed to Western blotting assay. WH performed the qRT assay. XH provided the clinical breast cancer specimens and performed IHC experiment. All authors discussed the results and edited this manuscript.

## ETHICS APPROVAL AND CONSENT TO PARTICIPATE

We declare that the study includes a statement on ethics approval and consent.

## CONSENT FOR PUBLICATION

Not applicable.

## Supporting information

Fig S1‐S3

Table S1

## Data Availability

All data generated or analysed during this study are included in this published article [and its supplementary information files].
