# Supplementary material for: Aberrant methylation of WD‐repeat protein 41 contributes to tumour progression in triple‐negative breast cancer
Source: J Cell Mol Med. 2020 May 12;24(12):6869–82. doi: 10.1111/jcmm.15344 (PMC7299681; doi:10.1111/jcmm.15344)
Supplement: Supplementary file 1 — Fig S1‐S3 [file JCMM-24-6869-s001.pdf]

Figure S1

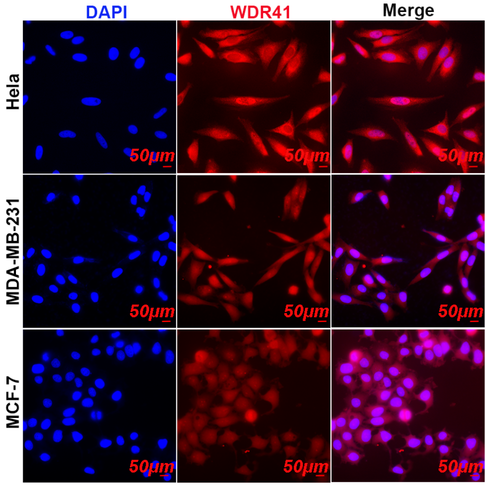

Supplementary Figure 1. Immunofluorescent staining of WDR41 in Hela, MDA-MB-231, and MCF-7 cells.

Figure S2

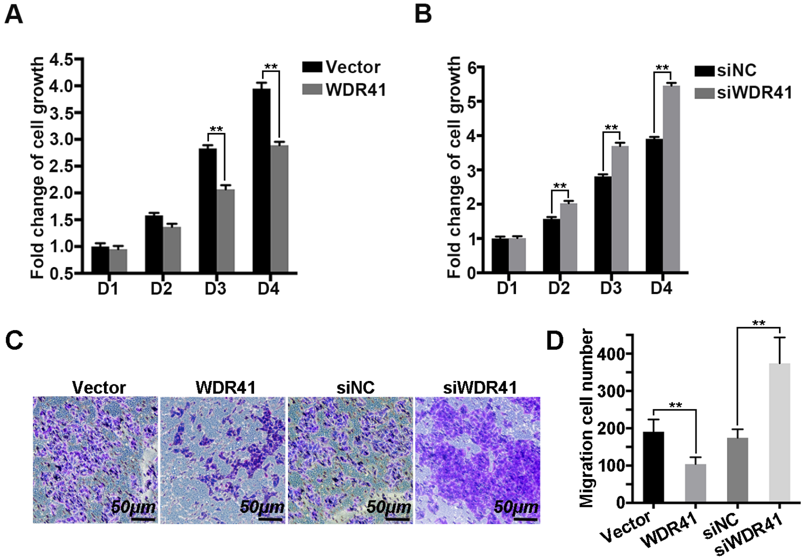

Supplementary Figure 2. Effect of WDR41 on cell growth and metastasis ability of BT549 cells. (A) and **(B)** MTT assay was performed to measure the cell growth ability in Vector, WDR41, siNC and siWDR41 group cells. **(C)** Trans-well assay is presented here in the above four different group cells. Scale bar, 50μm**.** **(D)** Average migration cell numbers of 5 HPFs in Vector, WDR41, siNC, and siWDR41 group. Vector, EGFP group (control); WDR41, EGFP-WDR41 group. siNC, negative control group; siWDR41, WDR41-siRNA group. ** $p<0.01$.

Figure S3

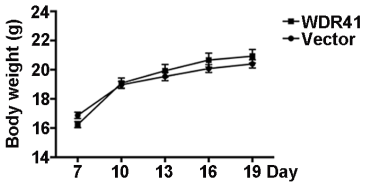

Supplementary Figure 3. Body weights in the xenograft model of Vector and WDR41 groups were measured during model build.
