# Supplementary material for: Aberrant methylation of WD‐repeat protein 41 contributes to tumour progression in triple‐negative breast cancer
Source: J Cell Mol Med. 2020 May 12;24(12):6869–82. doi: 10.1111/jcmm.15344 (PMC7299681; doi:10.1111/jcmm.15344)
Supplement: Supplementary file 2 — Table S1 [file JCMM-24-6869-s002.docx]

**Table S1 Primers for methylation specific PCR**

| **Gene** | **Left M Primer** | **Right M Primer** | **Left U Primer** | **Right U Primer** |
| --- | --- | --- | --- | --- |
| ***WDR41-1*** | TGTTATAGGGAGGGGTATTATTTTTC | ACGAAACTTAAATAACTCGAACGAC | TGTTATAGGGAGGGGTATTATTTTTT | ACAAAACTTAAATAACTCAAACAAC |
| ***WDR41-2*** | GTTATAGGGAGGGGTATTATTTTTC | ACGAAACTTAAATAACTCGAACGAC | TGTTATAGGGAGGGGTATTATTTTTT | ACAAAACTTAAATAACTCAAACAAC |
| ***WDR41-3*** | TGTTATAGGGAGGGGTATTATTTTTC | ACGAAACTTAAATAACTCGAACGAC | TGTTATAGGGAGGGGTATTATTTTTT | CACAAAACTTAAATAACTCAAACAAC |
| ***WDR41-4*** | GTTATAGGGAGGGGTATTATTTTTC | ACGAAACTTAAATAACTCGAACGAC | TGTTATAGGGAGGGGTATTATTTTTT | CACAAAACTTAAATAACTCAAACAAC |
| ***WDR41-5*** | TGTTATAGGGAGGGGTATTATTTTTC | ACGAAACTTAAATAACTCGAACGAC | TATAGGGAGGGGTATTATTTTTTGT | CAAAACTTAAATAACTCAAACAAC |
